# MDA-5 associated rapidly progressive interstitial lung disease with recurrent Pneumothoraces: a case report

**DOI:** 10.1186/s12890-018-0622-8

**Published:** 2018-04-17

**Authors:** Safi Alqatari, Peter Riddell, Sinead Harney, Michael Henry, Grainne Murphy

**Affiliations:** 1 Royal College of Physicians of Ireland, Rheumatology Department, Cork University Hospital, Wilton, Cork Ireland T12 DC4A; 2 Rheumatology Department, Cork University Hospital, Wilton, Cork Ireland; 3 Internal Medicine and Rheumatology Consultant Rheumatology Department, Cork University Hospital, Wilton, Cork Ireland

**Keywords:** Myositis, Interstitial lung disease, MDA-5

## Abstract

**Background:**

Clinically hypomyopathic dermatomyositis is a rare disease that is important to recognize, investigate and treat early as it is associated with poor prognosis. In a proportion of patients, myositis specific antibodies could be negative, but with high clinical suspicion, myositis associated antibodies should be ordered. Anti-MDA-5 antibodies was reported in literature to be associated with severe and rapidly progressive interstitial lung disease, with few case reports of pneumothorax and/or pneumomediastinum.

**Case presentation:**

A 49-year-old previously healthy lady, presented with a 6 week history of skin rash, photosensitivity, mouth ulcers, fatiguability, arthralgia and myalgia. She denied subjective weakness, respiratory symptoms or dysphagia. She had Raynaud’s phenomenon affecting her fingers only. Initial examination showed synovitis in her hands with skin rash. Autoimmune screen was negative. She was started on hydroxychloroquine. 4 weeks later on follow-up, she developed proximal muscle pain, dysphagia, dyspnea and dry cough. Examination showed mild proximal muscle weakness and bi-basal crackles. She was admitted and extended myositis screen was sent. She had mild anemia, lymphopenia and neutropenia, normal inflammatory markers, liver and renal panels. Capillaroscopy showed pattern of systemic sclerosis. CT chest showed early ILD. Electromyography and MRI showed features of mild myositis. PFT showed muscle weakness with low DLCO. She was given intravenous steroid and Rituximab. As she continued to deteriorate, intravenous immunoglobulins and cyclophosphamide were given. There was a brief clinical response that was short-lived with increasing oxygen dependency necessitating transfer to the ICU. At this point, the extended myositis screen confirmed the presence of anti-MDA-5 antibodies. She commenced plasmapharesis and required intubation. Unfortunately, she developed multiple pneumothoraces, and was transferred urgently for ECMO. Subsequent immunosuppression included rituximab and tacrolimus. There was progression of her ILD and recurrent pneumothoraces and pneumomediastinum. Unfortunately, she passed away as a consequence of her disease.

**Conclusion:**

This case highlights a number of considerations in approaching patients with inflammatory myositis, particularly to pulmonary involvement. It is important to highlight the utility of extended myositis antibody testing in predicting disease phenotypes and its impact on therapeutic decisions. From a management perspective, aggressive immunosuppression should be considered with potential need of earlier utilization of ECMO.

## Background

Clinically hypomyopathic DM is a rare disease that is important to recognize, investigate and treat early as it is associated with poor prognosis. In a proportion of patients, myositis specific antibodies are negative, but with high clinical suspicion, myositis associated antibodies should be ordered. Hypomyopathic dermatomyositis with positive anti-MDA-5 antibodies was reported in literature to be associated with severe and rapidly progressive interstitial lung disease. There are a limited number of case reports of associated pneumothorax and/or pneumomediastinum largely in an Asian population. We present a challenging case of rapidly progressive interstitial lung disease (ILD) and pneumothorax/pneumomediastinum in an Irish/Caucasian patient with hypomyopathic MDA-5 positive dermatomyositis.

## Case presentation

A 49-year-old lady was referred to the rheumatology services with a 6-week history of an erythematous rash on her face, fingers and feet which was painful, desquamative and itchy. This was photosensitive in nature and accompanied by painful mouth ulcers. More recently she had noticed generalized myalgia, widespread joint pain especially in her small joints, easy fatiguability and malaise. She reported symptoms of Raynaud’s phenomenon on exposure to cold two years prior. She denied respiratory symptoms, dysphagia or odynophagia. Examination was remarkable for puffy fingers with polyarthritis (MCPs, PIPs, DIPs, wrists and toes), peri-ungual erythema and a desquamative rash. Power on initial presentation was normal and there were no clinical findings to suggest ILD. She was commenced on low dose prednisolone and hydroxychloroquine and serological testing was requested. A standard screen was negative (RF, ACPA, ANA, ENA, ANCA). She had no family history of autoimmune disorders.

Over the ensuing 4 weeks she experienced a significant deterioration. She developed new progressive proximal muscle pain with no subjective weakness. She also reported dysphagia to solids as well as dyspnea on minimal exertion associated with a dry cough. Clinical examination revealed a mild proximal myopathy and end inspiratory bi-basal crackles. At that point, she was admitted for investigation and management including an extended myositis antibody panel. Results of her initial investigations are summarized in Table 1.

**Table 1:** Results of blood tests

Test	Result	Test	Result	Test	Result
WBCs	2.25/ L (4.4–11.3/L)	Urea	2.4 mmol/L (2.8–8.4 mmol/L)	Urinalysis	Negative
Neutrophils	1.2/ L (1.4–6.6/L)	Creatinine	54 micromol/L (49–90 micromol/L)	ANA and ENA	Negative
Lymphocytes	0.6/ L (0.9–3.2/L)	ALT	48 U/L (0–34 U/L)	DsDNA	Negative
Hemoglobin	10.1 g/dl (11.7–15.9 g/dl)	AST	78 U/L (6–42 U/L)	C3	1.15 g/L (normal)
Platelets	420,000/L (140–440 X10*9/L	LDH	1531 U/L (220–450 U/L)	C4	0.30 g/L (normal)
ESR	7 mm (0–20 mm)	CPK	208 U/L (40–180 U/L)	RF and Anti-CCP	Negative
CRP	2 mg/L (0–10 mg/L)	Albumin	25 g/L (35–52 g/L)	ANCA	Negative

**Fig. 1:**
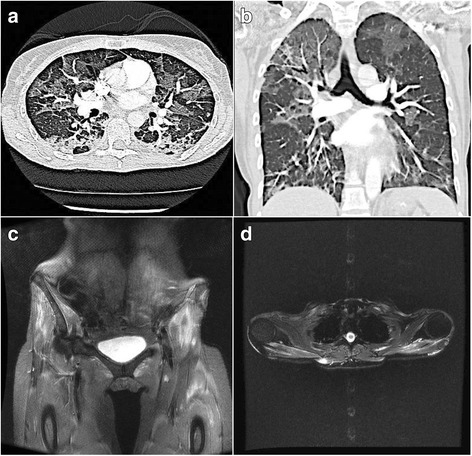
Axial (**a**) and Coronal (**b**) CT (lung windows). Multifocal ground glass opacity progressing to consolidation in the dependent lungs. (**d**) Coronal STIR Pelvis: T2 hyperintensity in gluteal, quadriceps and adductor musculature in keeping with myositis. (**e**) Axial T2FS Upper limbs: Further oedema within trapezius, deltoid and rotator cuff musculature

Echocardiography was normal. Capillaroscopy showed early and active pattern of systemic sclerosis. CT chest showed early ILD in the form of non-specific interstitial pneumonia (NSIP) (Fig. 1a, b). Electromyography showed features of mild non-necrotic myopathy in the quadriceps muscles. MRI of bilateral humeri and femora showed features of myositis involving multiple muscle groups (Fig. 1c, d). PFT showed restrictive pattern with moderate DLCO reduction (DLCO 11.8 (predicted = 20), FEV1 1.7 L (predicted = 2.02), FVC 2.1 (predicted = 2.4), FEV//FVC = 80.95%).

She was commenced on methylprednisolone 500 mg intravenously for 3 days and received Day 1 Rituximab 1 g, with a plan to retreat on Day 14. Unfortunately, she developed a steroid-induced psychosis, necessitating a reduction in subsequent prednisolone dosing. Over the ensuing days she further deteriorated with radiological progression of her ILD. In the context of progressive dysphagia, she commenced IV Immunoglobulin and received cyclophosphamide (15 mg/kg). There was a brief clinical response following this combination but this was short-lived with increasing oxygen dependency necessitating transfer to the ICU for initial non-invasive ventilation. At this point, the extended myositis screen detected the presence of anti-MDA-5 antibodies confirming the diagnosis of hypomyopathic dermatomyositis. She commenced plasmapharesis for 5 days. She required intubation due to respiratory fatigue and increasing oxygen requirements. She developed multiple pneumothoraces, and due to ventilatory difficulties was transferred urgently for ECMO. Subsequent immunosuppression included day 14 rituximab and the addition of tacrolimus. Despite aggressive immunosuppression and prolonged ECMO therapy there was further progression of her interstitial lung disease, recurrent pneumothoraces and pneumomediastinum. Unfortunately, she passed away as a consequence of her disease.

## Discussion and conclusion

Idiopathic inflammatory myopathies (IIMs) are a heterogeneous group of diseases [polymyositis (PM), dermatomyositis (DM), inclusion body myositis (IBM), necrotizing autoimmune myositis (NAIM)], characterized by myositis. Other organs such as the skin in DM and the lungs, heart, joints and gastrointestinal tract can be affected. Classification of IIMs is important as different phenotypes and serotypes are caused by different presentations, suggesting the need for targeted therapies [1]. Autoantibody testing has become crucial for the assessment of patients with IIM (as described by Betteridge and McHugh) [2].

Auto-antibodies can be generally classified into myositis-associated and myositis-specific antibodies. The recognition of specific clinical phenotypes associated with individual antibodies has had significant clinical impact. Notable examples include the association of antibodies directed at TIF-1gamma with malignancy. Antisynthetase antibodies are strongly associated with ILD, especially non-Jo-1 (anti-PL-7 and anti-PL-12) where the association approaches 90–100% [3]. A rare form of ILD with a very poor prognosis, is strongly associated with CADM and the novel MDA- 5/anti-CADM-140 auto-antibody. A combination of anti-Jo-1 and anti-SSA/Ro antibodies also indicates severe pulmonary involvement [3].

The strong association between ILD and myositis has been recognized for many years and has led to the routine screening for ILD in individuals presenting with an inflammatory myopathy. Clinically amyopathic or hypomyopathic dermatomyositis however may initially present to a variety of specialties and ultimately this can lead to diagnostic delay. These individuals may present initially to respiratory services with symptoms and signs of ILD. The advent of EMA testing has suggested that particular antibodies segregate with propensity for ILD, such as PL-7 and PL-12. Interestingly these individuals who may present with imaging in keeping with established fibrosis may respond to immunosuppression [3]; highlighting the importance of requesting extended antibody testing, even in the absence of circulating anti-nuclear antibodies. In the case of MDA-5 antibodies in particular, ILD can be rapidly progressive and, as in the case discussed above, refractory to intensive immunosuppression, suggesting its valuable utility as a prognostic indicator of disease severity.

In our case, it is probably the high pressure from ventilatory support that caused the pneumothoraces but it is important to highlight an interesting and perhaps less recognized phenomenon, which is the potential for pneumothoraces and pneumomediastinum. Both can occur spontaneously as published in previous case reports/series [4]. The aetiopathogeneis is poorly understood but multiple theories have been proposed. These include the presence of raised intra-alveolar pressure which leads to rupture of previously damaged alveoli, or rupture of subpleural cyst(s) that developed from co-existing interstitial fibrosis. Another factor could be the presence of an underlying lung vasculitis, as DM is associated with inflammation of small blood vessels [4]*.* Many patients with rapidly progressive ILD and amyopathic myositis needed a combination of multiple immunosuppressives and steroids for a prolonged period (up to twelve months in some reports*)* highlighting the aggressive nature of ILD in this cohort.

Given the rarity of individuals with myositis associated antibodies, trials aiming to target a particular therapy by antibody type do not exist. Existing reports suggest that initial high dose steroids are the most common initial therapy used and perhaps the most effective medication in controlling acute disease. They are used in conjunction with a variety of additional immunosuppressives including cyclophosphamide, mycophenolate mofetil, ciclosporin and tacrolimus and biologic medication, commonly rituximab. Of note, rituximab offers dual benefit in treating accompanying myopathy and is also gaining increasing attention in treating ILD in the context of connective tissue disorders. Some studies suggested that the addition of tacrolimus improved the disease free survival of PM/DM as will as ILD. However, the number of patients involved in those studies were small [5].

Finally, the impact of ECMO therapy merits discussion and particularly its role in reducing the risk of pneumothorax in this setting. In the case of rapidly progressive ILD associated with CADM, the use of ECMO should be considered early in the disease process, especially in potentially reversible conditions to avoid the need for mechanical ventilation [6].

In conclusion, this case highlights a number of critical considerations in approaching patients with inflammatory myositis, particularly in relation to associated pulmonary involvement. While ILD has long been recognized as a potential complication of myositis, the potential for ILD to be the prominent feature of presentation is now well described particularly in a subgroup of patients with little clinical evidence of myopathy. We wish to highlight the importance of clinical surveillance for the development of myopathy in individuals with amyopathic or hypomyopathic dermatomyositis; the utility of extended myositis antibody testing in predicting disease phenotypes and the potential impact on subsequent management in this cohort. Of particular significance, this case emphasized the importance of early consideration of ECMO to decrease the risk of pneumothorax/ mediastinum.

## Abbreviations

ACPA: Anti-cetrullinated peptide antibody
ANA: Anti-nuclear antibody
ANCA: Anti-neutrophilic cytoplasmic antibody
CADM: Clinically amyopathic dermatomyositis
DIPs: Distal inter-phalangeal joints
DLCO: Diffusion carbon monoxide
DM: Dermatomyositis
ECMO: Extra-corporeal membrane oxygenation
ENA: Extractable nuclear antibody
FEV1: Forced expiratory volume in 1 s
FVC: Forced vital capacity
IBM: Inclusion body myositis
IIMs: Idiopathic interstitial myopathies
ILD: Interstitial lung disease
MCPs: Meta-carpo-phalangeal joints
MDA-5: melanoma differentiation-associated gene 5
MRI: Magnetic resonance imaging
NAIM: Necrotizing autoimmune myositis
NSIP: Non-specific interstitial pneumonia
PFT: Pulmonary function test
PIPs: Proximal inter-phalangeal joints
Anti-PL-7: Anti-Threonyl-tRNA Synthetase
Anti-PL-12: anti-alanyl-tRNA synthetase
PM: Polymyositis
RF: Rheumatoid factor
